# Recovery index, attentiveness and state of memory after xenon or isoflurane anaesthesia: a randomized controlled trial

**DOI:** 10.1186/1471-2253-10-5

**Published:** 2010-05-07

**Authors:** Ralph Stuttmann, Jens Jakubetz, Kati Schultz, Claudia Schäfer, Sebastian Langer, Utz Ullmann, Peter Hilbert

**Affiliations:** 1 Department of Anaesthesiology/Intensive Care and Emergency Medicine/Pain Therapy, BG-Kliniken Bergmannstrost, (Merseburger Strasse 165), Halle/Saale, (06112), Germany; 2 Department of Psychology, BG-Kliniken Bergmannstrost, (Merseburger Strasse 165), Halle/Saale, (06112), Germany

## Abstract

**Background:**

Performance of patients immediately after anaesthesia is an area of special interest and so a clinical trial was conducted to compare Xenon with Isoflurane anaesthesia. In order to assess the early cognitive recovery the syndrome short test (SST) according to Erzigkeit (Geromed GmbH) was applied.

**Methods:**

ASA I and II patients undergoing long and short surgical interventions were randomised to receive either general anaesthesia with Xenon or Isoflurane. The primary endpoint was the validated SST which covering memory disturbances and attentiveness. The test was used on the day prior to intervention, one and three hours post extubation. The secondary endpoint was the recovery index (RI) measured after the end of the inhalation of Xenon or Isoflurane. In addition the Aldrete score was evaluated up to 180 min. On the first post-operative day the patients rated the quality of the anaesthetic using a scoring system from 1-6.

**Results:**

The demographics of the groups were similar. The sum score of the SST delivered a clear trend one hour post extubation and a statistically significant superiority for Xenon three hours post extubation (p < 0.01). The RI likewise revealed a statistically significant superiority of Xenon 5 minutes post extubation (p < 0.01). The Aldrete score was significantly higher for 45 min. The scoring system results were also better after Xenon anaesthesia (p < 0.001).

**Conclusions:**

The results show that recovery from anaesthesia and the early return of post-operative cognitive functions are significantly better after Xenon anaesthesia compared to Isoflurane. The results of the RI for Xenon are similar with the previously published results.

**Trial Registration:**

The trial was registered with the number ISRCTN01110844 http://www.controlled-trials.com/isrctn/pf/01110844.

## Background

In recent years several clinical trials have been published that characterize the anaesthetic properties as well as the other potential properties of Xenon [1-4]. Following the licensing of Xenon in 12 European countries in 2007 after Germany was first in 2005 there is increasing interest in gaining a better understanding of the underlying scientific opportunities that Xenon offers as well as the role that Xenon can play in anaesthesia in the future.

In 1951 Cullen [5] performed an inhalational anaesthesia with Xenon in old patients and showed excellence results regarding recovery and memory. There is a lack of data pertaining to healthy patients and recovery of post-operative cognitive functions such as attentiveness and memory particularly with regard to recovery on the day of surgery following use of Xenon anaesthesia versus standard inhalational anaesthesia. To demonstrate an advantage of Xenon on emergence from anaesthesia and early post-operative recovery of cognitive functions assessed as attentiveness and memory an appropriate method is necessary. The syndrome short test (henceforth referred to as SST) according to Erzigkeit (Geromed GmbH) has proven to be reliable for more than 15 years, it is suitable for use in the first hours after extubation, easy to handle, easy to use repeatedly and to replicate [6-11] The aim of this study was to investigate the emergence from inhalational anaesthesia using either Xenon or Isoflurane until extubation time; and subsequently to evaluate early recovery of attentiveness and memory using the SST.

## Methods

This was a prospective, randomised phase III clinical trial, single blind with two parallel groups. The study protocol was approved by the ethics committee of the University Witten/Herdecke (session nr. 23/2002 of 11.09.2002; patient insurance by Winterthur International Nr. DE 00020283-LI-02A-715) and prior to inclusion patients had to give their written consent.

### Hypotheses

The aim of the study was to evaluate the efficacy of two balanced anaesthetic regimens with respect to recovery of early post-operative cognitive function at the day of operation (assessed as attentiveness and memory; this was the primary criterion) and emergence profile (assessed as the recovery index; this was the secondary criterion). The hypothesis was primarily that patients have a significantly higher status of attentiveness and memory 1 and 3 hours after inhalation anaesthesia with Xenon compared to Isoflurane anaesthesia, and that patients reach their pre-operative level of cognitive function within 3 hours after extubation. The second hypotheses was, that the time to wake up from the anaesthetic was predicted to be shorter following Xenon than following Isoflurane.

### Test instruments Syndrome Short Test (SST) and Recovery Index (RI)

The SST was provided by Geromed GmbH (address - Cadolzburgerstr, 6, D-91074 Herzogenaurach, Germany) and consists of nine subtests - 1 naming objects, 2 the immediate reproduction of objects, 3 reading numbers, 4 sorting numbers, 5 counting backwards, 6 counting symbols, 7 an interference test, 8 reproduce objects and 9 recognising objects. Each subtest can take up to 60 seconds. Subtests 2, 8 & 9 evaluate memory and subtests 1, 3, 4, 5, 6 & 7 evaluate attentiveness. The time taken to run the tests and the number of mistakes made in the subtests were documented on an interpretation sheet; points were allocated adjusted for age and sex. In the case of a homogeneous distribution of the number of points in the two subtests, i.e., memory (subtests 2, 8, 9) and attentiveness (subtests 1, 3, 4, 5, 6, 7) the sum score can be used to describe the result; if the distribution is not homogeneous then the sum score of the subtests for memory and attentiveness have to be used. To carry out tests for homogeneous or non homogeneous distribution of the results a special graphic sheet was developed by Erzigkeit [10]. The test system is suitable for repetition and held in the hospital in two different versions to avoid familiarisation. Within the frame of this study the baseline results were taken pre-operatively (on the day before operation) and post-operatively at 60 and 180 minutes post extubation. The investigator measuring the SST pre- and post-operatively was a medical assistant and was blinded for the inhalational anaesthetic utilized.

While the early post-operative cognitive recovery was evaluated by means of the SST the recovery profile of anaesthesia with respect to time until extubation and general function was evaluated by the validated recovery index[12]. The recovery index (RI) is defined as a balanced ratio between the Aldrete score [13] measured 5 minutes post extubation plus 1 divided by the weighed extubation time (+2) and the elapsed time until opening eyes (+1), both measurements beginning at the end of anaesthesia and withdrawal of the anaesthetics. Three of the doctors in the department were responsible for conducting the anaesthesia and were also responsible for measuring eye opening, extubation time and all other intra-operative data.

### Patients

Healthy ASA I and II patients over 18 years of age scheduled for four different types of elective surgery were eligible. The types of surgery were: visceral surgical strumectomy, augmentation or reduction mammaplasty, liposuction in obese patients and knee arthroscopy. Patients were randomised in variable blocks of 4-8 patients in order to balance the groups using simple self-programmed software. The varying size of blocks was to counteract the differing frequencies of the operations and also any influence of duration and invasiveness of the operations. A total of 61 patients who met the pre-defined inclusion and exclusion criteria were randomised either to receive Xenon in oxygen (Xenon group, n = 31) or Isoflurane in stickoxydul (Isoflurane group (n = 30). A power analysis was calculated for 30 patients in each group to show a difference of one SD with a probability of 96% and a difference of 0.75 SD with a probability of 82% (alpha = 0.05).

### Anaesthesia

No premedication was administered prior to induction. All patients were monitored with ECG leads, repeated non invasive blood pressure measurement and oxygen saturations. In both groups induction was accomplished by intravenous injection of 1-2 mg/kg bodyweight (bw) Propofol, 0.003 mg/kgbw Fentanyl and 0.6 mg/kgbw Rocuronium Bromide, to facilitate intubation. Patients in the liposuction group additionally had tumescence with adrenalin with a concentration of 0,66 mg/1000 ml and a volume of 1-2 litres dependent on the field of surgery. In the induction room and also for the first time in the operating room the maintenance of anaesthesia was achieved with continuously infused Propofol and, if necessary, by repeated increments of Fentanyl (0.0015 mg/kgbw). Rocuronium Bromide was not repeated during the operation.

In the operating room the Physioflex ventilator (closed system) with the Xenon option was used (Dräger Company, Germany). All patients were volume-controlled (7 ml/kgbw) and time-circled ventilated with identical ventilation settings on a closed system until extubation without spontaneous breathing. The target concentration of end-tidal CO2 was adjusted to 35 mmHg. After reaching a target concentration for oxygen of 98%-100% the oxygen concentration was lowered to 30% in both groups to start the administration of either Xenon or Isoflurane in N_2_O (end-tidal concentration 63-65%). Normally at this point the surgeons prepared the surgical site by disinfection meaning that all patients were in a stable steady state of anaesthesia at incision and during any surgical stimulation.

In the case that during anaesthesia the systolic BP dropped below 100 mmHg for more than5 minutes the patients recieved Akrinor^® ^(a mixture of Cafedrin and Theoadrenalin) on demand.

In the Xenon group, Xenon was flushed in by repeated use of the flush button to reach the end-tidal target concentration of approximately 63% within a span of several minutes; the Propofol infusion was then stopped. If the Xenon concentration fell below 58% during operation then the flush button was repeatedly used to augment Xenon concentration in the gas mixture until a Xenon concentration of 60% or more was achieved again. The procedure in the Isoflurane group was identical - the inspired oxygen concentration of 98-100% was lowered to 30% and N_2_O was also flushed in to lower the MAC. Simultaneously Isoflurane was given to each patient with an end-tidal concentration of 0.6 vol%. The Propofol infusion was also stopped at this point. If signs occur that the patient was too lightly anaesthetised despite a sufficient inspiratory concentration of Xenon or Isoflurane, Fentanyl was administered in increments of 0.003 mg/kgbw to deepen anaesthesia. Depth of anaesthesia was recorded with the bispectral index monitoring system (BIS, software 2.21; Aspect Corporation). The BIS index is widely used to monitor the depth of anaesthesia [14]. A BIS value of 40 ± 10 was accepted as sufficient in both groups. Additional application of Fentanyl increments was only orientated by clinical signs of too light anaesthesia and independent of the index number indicated by the BIS monitor as the BIS monitor is not validated for use with Xenon. As expected the duration of anaesthesia in the present study differed with the different surgical interventions however, a balanced number of cases per type of intervention by randomisation in blocks helped to reduce this influence.

At the end of operation with all surgical interventions finished the Isoflurane vaporizer was set to zero vol% and simultaneously the breathing system flushed with oxygen to eliminate both inhalational anaesthetics. In the Xenon group the breathing system was also flushed with oxygen to eliminate Xenon at the same point. The time at which this occurred was taken in both groups to be the end of anaesthesia. The patients were asked every minute to open their eyes and to follow commands. Extubation was performed at the time of eye opening provided that there was spontaneous breathing and that the patient was otherwise stable. Eye opening time and extubation time were noted. Five minutes post extubation the Aldrete score was evaluated for the first time. Further evaluation of the Aldrete score was performed at 15, 30, 45, 60, 90, 120 and 180 minutes post extubation to measure general performance. On the first post-operative day all patients were visited by the person measuring the SST and asked to judge the quality of anaesthesia by using a simple scoring system - a numerical value between 1 and 6 with 1 for poor and 6 for excellent.

Additional documented parameters covered blood pressure, heart rate, oxygen saturation and laboratory data as necessary. Different times of anaesthesia, the Xenon or Isoflurane inhalation time, the incision to suture time and the time of cessation of anaesthesia (flushing the breathing system of the Physioflex apparatus with oxygen) were documented.

The most common side effect of anaesthesia in the recovery room PONV was documented and treated with metoclopramide, dimenhydrinate and dolasetrone if necessary.

In the recovery room the patients received piritramide and metamizole on demand for pain relief.

### Statistical Analysis

Statistical analysis was performed with the Sigma Stat package (Systat software, Inc. version 3.5) after data entry and verification thereof. Sigma Plot (version 9.0) was used for graphical presentations.

Quantitative data were summarized by descriptive statistics - valid numbers, arithmetic means, medians, standard deviation, standard error, confidence intervals and box plots. Besides this, the nominal and ordinal scale data was presented as frequencies and corresponding percentages.

Two Way Analyses of Variance were used in the case of normal distributions. In case of abnormal distributions appropriate non-parametric tests were used. Taking scale levels into account possible inter group differences were evaluated with the Wilcoxon-Mann-Whitney-U-Test, Wilcoxon-Pratt test and the Mantel-Haenszel Test. Values of p < 0.05 (α = 5%: How to reject the hypothesis of no difference) were considered as statistically significant. The primary endpoint only (SST: 3 hours post intervention) was interpreted as confirmatory. The sum test result of the SST had to be proven for homogeneity. In case of non homogeneous distribution between the subtests for memory and attention the summarized sub tests were compared between each group.

## Results

Key demographic data of the patients are described in Table 1 - as shown in this table both groups were comparable. Due to low patient numbers in the subgroups a consolidation of the data divided into clinical indications was indicated for statistical calculations. The conduction of anaesthesia was uneventful in both groups. The circulatory parameters and the BIS value are only evaluated during operation and reflect the time of Xenon insufflations. The circulatory data showed the advantages of the Xenon anaesthesia with a lower heart rate and a higher mean arterial pressure (MAP). The systolic and diastolic pressures were also higher in the Xenon group. The BIS value was always within the desired level and nearly identical in both groups except that after the start of Xenon insufflations the BIS level was slightly lower in the Xenon group for only ten minutes - it is unlikely that this difference has influenced the wake up time. Indeed the circulatory data and the BIS level are interpreted as a stable depth of anaesthesia and no patient reported awareness on the visit one day post-operatively.

**Table 1:** Demographic data: Xenon and Isoflurane anaesthesia.

	Xenon	Isoflurane
n	31	30
Age [years]	41,5 (CI95: 36.4, 46.7)	38.9 (CI95: 34.0. 43.8)
Gender [m/f]	8/23	6/24
**ASA class**		
I	10/(32.3%)	7/(23.3%)
II	21/(67.7%)	23/(76.7%)
Height [cm]	166.9 (CI95: 164.4, .69.4)	169.4 (CI95: 166.0. 70.2)
Body weight [kg]	68.2 (CI95: 63.8, 72.6)	71.2 (CI95: 66.2, 76.3)
		
**Surgical intervention/incision to suture time (minutes)**		
Visceral strumectomy [n]	9/124.9 SD 28.1	9/102.7 SD 27.8
Knee arthroscopy [n]	7/74.7 SD 56.2	7/105.0 SD 68.2
Liposuction [n]	8/194.9 SD 83	8/199.1 SD 33.5
Mammaplasty [n]	7/119.1 SD 27	6/129.7 SD 68.2

Regarding the drug consumption during surgery, additional medication in the recovery room and for pain relief we refer to Table 2. PONV occurs in 13 patients in the Xenon group and in 8 patients in the Isoflurane group. One patient in the Isoflurane group received Akrinor^® ^due to low blood pressure.

**Table 2:** Comparison regarding Drug consumption in both groups.

Drug	Xenon	Isoflurane
Propofol total consumption	219.5 mg (SD 54.49) n = 31	190 mg (SD 42) n = 30

Fentanyl for induction	0.273 mg (SD0.05) n = 31	0.286 mg (SD 0,048) n = 30

Fentanyl consumption during surgical procedure	0.46 mg (SD 0.23) n = 31	0.216 mg (SD 0.17) n = 30

Rocuronium total consumption	39.4 mg (SD 7,5) n = 31	40.6 mg (SD 8.6) n = 30

Piritramide	5.9 mg (SD3.2) n = 7	7.8 mg (SD 2.7) n = 14

Metamizole	1 g n = 1	0

Dimenhydrinate	82.7 mg (SD 35.8) n = 3	31 mg n = 1

Metoclopramide	10 mg (SD 0) n = 12	13.7 mg (SD 5) n = 6

Dolasetrone	13.9 mg (SD 4.2) n = 9	10.8 mg (SD2.9) n = 3

### Recovery Index

**Figure 1:**
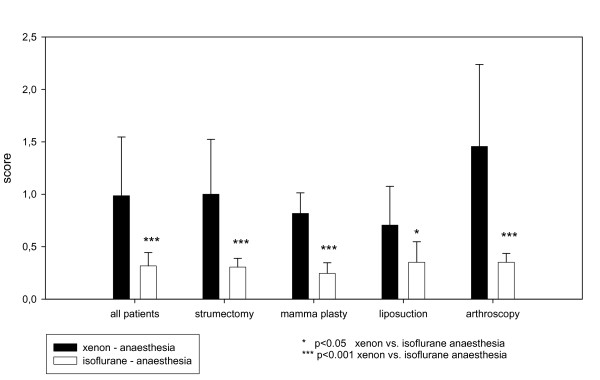
**Mean Recovery Index**.

In accordance with the previous findings the results of the post-operative RI demonstrated a clear superiority of Xenon in comparison with Isoflurane (Xenon: RI 1.0 SD 0.6; CI95: 0.8-1.2; Isoflurane: RI 0.3 SD 0.1; CI95: 0.2-0.4) which was confirmed by a statistical significant and clinically relevant difference (P < 0.01). These results are visualized in Figure 1 and all the data used to calculate the RI is in Table 3.

**Table 3:** Eyes opening time, extubation time and Aldrete score after 5 min.

Group	All	strumectomy	mammaplasty	liposuction	arthroscopy
**Xenon **(eyes opening time)					

N	31	9	7	8	7

Mean	4,0	4,0	4,1	4,8	2,9

SD	2,0	2,2	1,6	2,3	1,6

CI	3,3 -- 4,7	2,3 -- 5,7	2,6 -- 5,6	2,9 -- 6,7	1,4 -- 4,4

**Isoflurane **(eyes opening time)					

N	30	9	6	8	7

Mean	8,9	8,1	11,7	9,1	7,1

SD	3,5	2,9	4,3	3,9	2,0

CI	7,6 -- 10.2	5,9 -- 10.3	7,2 -- 16,2	5,8 -- 12,4	5,3 -- 8,9

**Xenon **(extubation time)					

N	31	9	7	8	7

Mean	4,5	4,2	4,7	5,5	3,3

SD	2,2	2,4	1,1	2,8	1,9

CI	3,7 -- 5,3	2,4 -- 6,0	3,7 -- 5,7	3,1 -- 7,9	1,6 -- 5,0

**Isoflurane **(extubation time)					

N	30	9	6	8	7

Mean	10.2	9,6	12,8	10.0	9,0

SD	3,5	2,7	4,4	4,2	2,2

CI	8,9 -- 11,5	7,5 -- 11,7	8,2 -- 17,4	6,5 -- 13,5	7,0 -- 11,0

**Xenon **(Aldrete score, 5 minutes post extubation)					

N	31	9	7	8	7

Mean	8,8	8,4	9,6	7,9	9,7

SD	1,1	0.5	0.5	1,4	0.8

CI	8,4 -- 9,2	8,0 - 8,8	9,1 -- 10.1	6,8 - 9,0	9,0 -- 10.4

**Isoflurane **(Aldrete score, 5 minutes post extubation)					

N	30	9	6	8	7

Mean	7,3	6,9	7,5	7,5	7,6

SD	1,2	1,5	1,2	1,4	0.8

CI	6,8 - 7,8	5,8 -- 8,0	6,2 -- 8,8	6,3 -- 8,7	6,9 -- 8,3

Xenon versus Isoflurane anaesthesia (Mean, SD, CI).

### Syndrome short test

An SST with a sum score of 0-2 points is representative for healthy people without any disturbance of cognitive function; a test result of 3-4 points indicates suspicion of cognitive dysfunction; 5-8 points suggests light cognitive dysfunction and 9-13 points suggests the beginning of a psycho-organic syndrome. The results of the pre-operative measurement of the SST were normal and comparable in both groups with a mean of 1.5 points (CI: 0.8-2.2 points) in the Xenon group and 1.7 points (CI: 1.0-2.4 points) in the Isoflurane group. Post-operatively, the results of the SST suggested a superiority of Xenon in comparison with Isoflurane with the 1-hour results showing an apparent advantage of Xenon; this, however, could not be verified from a statistical point of view (Xenon SST after 1 h: 6.8 points; CI95: 5.2-8.4 points; Isoflurane SST after 1 h: 8.3 points; CI95: 7.0-9.6 points). These results and the clinical condition at 1 hour suggest light cognitive dysfunction. After 3 hours the results of the SST were continuously better in both groups and representative for healthy people without disturbance of cognitive function in the xenon group and the suspicion of cognitive dysfunction after Isoflurane. The difference was statistically significant in favour of Xenon (Xenon SST after 3 h: 2.1 points; CI95: 1.4-2.8 points; Isoflurane SST after 3 h: 3.8 points; CI95: 2.8-4.8 points) (P < 0.001).

The differentiation into the two subtests of the SST (memory and attentiveness) showed an expected deterioration in both groups between the pre-operative situation and the one-hour post extubation control. The three-hour post extubation results indicated an obvious trend to the pre-operative level in both groups in favour of Xenon. Neither the difference in the number of points in the subtests of memory nor in the subtests of attentiveness could reach statistical significance in any group (α = 0.05). At time spans of 1 and 3 hours, 7 patients revealed heavy or very heavy disturbance of memory in the Isoflurane group compared to only 2 patients in the Xenon group (not significant). At the same times the tests of disturbance of attention showed no difference in the number of patients with light and mean deteriorations (11 patients after Xenon and 13 patients after Isoflurane). These results are summarized in Table 4.

**Table 4:** Subscores of memory and attentiveness at three time points.

Disturbance of memory						
**Points**	**0--1**	**2--3**	**4--5**	**6--7**	**8--9**	

	**No**	**Light**	**mean**	**Heavy**	**very heavy**	

time	Xe/iso	xe/iso	xe/iso	xe/iso	xe/iso	Σ

Pre	28/28	1/2	2/0	0	0	31/30

1 h	9/13	9/3	11/9	2/4	0/1	31/30

3 h	24/21	7/4	0/3	0/2	0/0	31/30

						

Disturbance of attention						

Points	0--2	3--5	6--9	10--12	13--15	

	No	very light	light	mean	heavy	

time	xe/iso	xe/iso	xe/iso	xe/iso	xe/iso	Σ

Pre	26/24	5/5	2/1	0/0	0/0	31/30

1 h	7/8	12/12	9/7	3/3	0/0	31/30

3 h	24/19	5/8	2/3	0/0	0/0	31/30

Preoperatively (pre) after 1 and 3 hours. Xenon versus Isoflurane anaesthesia (xe/iso).

### Aldrete score

**Figure 2:**
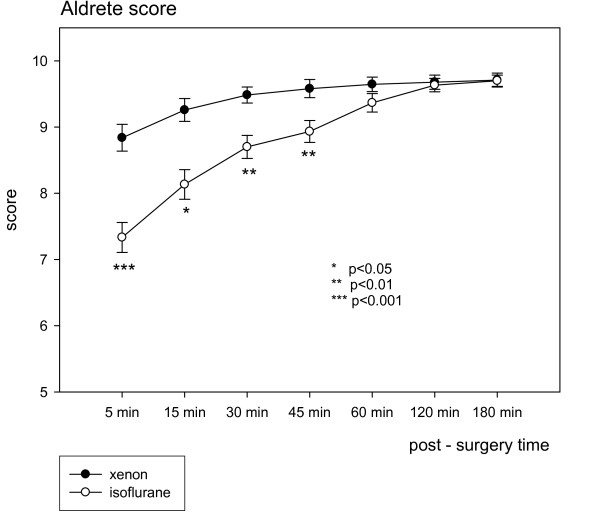
**Aldrete score at different time points**. Xenon respectively Isoflurane anaesthesia, all patients.

Post-operative the Aldrete score demonstrated a significant advantage in the Xenon group for 45 minutes and a positive trend for 60 minutes (Figure 2). Compared to the Isoflurane group in the Xenon group the maximum was reached earlier. Only liposuction patients with long incision to suture time showed no difference. Simple scoring system to assess the quality of anaesthesia by the patients post-operatively in the Xenon group were significantly better; i.e., mark 5 = very good. The corresponding mark in the Isoflurane group was 4 (P < 0.0005).

## Discussion

In hospital bedside care is facilitated on those patients who have a high level of both independence and cooperation.

The results of this clinical trial are focused firstly on the comparison of both patient groups with the SST. Secondly, the RI quotient using eye opening time, extubation time and Aldrete score was calculated. Both criteria are considered to be valuable tools to make an assessment of the recovery from anaesthesia during the first hours. While the RI provides information about the waking time directly after the end of anaesthesia, the SST generates very important information about parameters of cognitive function. The SST was measured at 1 and 3 hours post extubation to evaluate the early post extubation phase in healthy patients without any risk to develop cognitive dysfunction after anaesthesia and surgery. With these instruments the description of Cullen and the relation between the speed of recovery after the end of anaesthesia (RI) and the cognitive performance (SST) the first 3 hours there after has been proven.

In a review article Guinta [15] described the anaesthetic and pharmacologic properties of the NMDA receptor antagonist Xenon and N2O. Xenon has a blood-gas distribution coefficient of 0.14 and Isoflurane a higher blood-gas distribution coefficient of 1.41. Xenon has an oil-gas distribution coefficient of 1.9 and Isoflurane a much higher oil-gas distribution coefficient of 98. The oil-gas distribution coefficient (or fat solubility) determines the effectiveness of narcotic gases and influences the kinetics of distribution in the body. The oil-gas distribution coefficient of Xenon is associated with a better solubility in oil as compared with blood by a factor of 14 and for Isoflurane by a factor of 86. The smaller the factor the faster is the wake up time from anaesthesia. According to these data and the expected difference in the wake up time and the recovery of attentiveness and memory the sample size was calculated to be adequate to show a difference in the SST, when Xenon is compared to Isoflurane. Isoflurane is a widespread used inhalation anaesthetic and ideal for totally closed systems because the accumulation of potentially toxic compounds is unknown.

The minimal alveolar concentration (MAC) of Xenon was first described to be 0.71 (= 71%) [16]. More recently performed investigations corrected the MAC-value to 63%[17]. Following the SPC in Europe inhalation anaesthesia with Xenon has to be combined with an opioid. The target concentrations of Xenon of about 63% (MAC 1.0) and the combination with Fentanyl were in accordance with those recommendations. Isoflurane anaesthesia was combined with the NMDA receptor antagonist N2O as the porter gas *to *mimic the NMDA receptor antagonist Xenon and to lower the MAC and also supplemented with increments of Fentanyl. The depth of anaesthesia was measured by means of the BIS. The BIS monitor is empirically validated for anaesthetics drugs acting on the GABA receptor and was not yet validated for NMDA receptor antagonists like Xenon during the study periode. Thus, the depth of anaesthesia was controlled in both groups only by clinical signs. According to Goto [18], the BIS monitoring was also not used for the waking period because in patients waking up from Xenon anaesthesia the time to react to verbal commands can not be predicted by the BIS level. An influence on recovery by the invasiveness, frequency and duration of the intervention was excluded by a block randomisation.

The "recovery index" is deemed to be validated with data from the literature[19,20] The expressiveness of this index was verified in two large randomised clinical trials. A difference of the RI of 0.17 is classified as clinically relevant. In the investigation presented here the difference was 0.7 and therefore even distinctly more pronounced as shown in an arrangement of Xenon and Sufentanil being 0.3 [21]. All individual parameters shown in Table 3 as well as the index itself were significantly superior for Xenon as compared to the comparator Isoflurane.

The SST system was primary developed to test memory and attentiveness in elderly patients to detect dementia [22]; later on the test system was also used to describe the conditions of recovery after anaesthesia [23,24]. The use of the SST as the best suitable test system was carefully selected between other test systems in this study to investigate the early cognitive recovery, especially memory and attentiveness after general anaesthesia at the day of the operation [11,25,26]. Pre-operatively the mean value of the sum score of the SST was normal and within the same range in both groups. One hour post extubation, the patients showed a light disturbance of cognitive function as indicated by the sum score but there was a trend in favour of Xenon. No difference between the groups was observed in the sub-score for attention. In the sub-score for memory in the Isoflurane group 7 patients had heavy or very heavy disturbance of memory and only two patients in the Xenon group. After 3 hours the SST sum score was significantly better in the Xenon group and approximately the same compared to the pre-operative situation. At the same point in time the SST sum score in the Isoflurane group, was still in a range with suspicion of disturbance of cognitive functions. This is the first controlled randomised clinical trial showing a significant advantage of Xenon anaesthesia on the recovery profile of mental functions with an appropriate test instrument. Regarding the clinical daily life, the fast recovery after a Xenon anaesthesia reflects a good and normal response of the patient and a good restoration of memory equivalent to the pre-operative level already three hours after extubation.

Different clinical trials which investigate how patients recover and wake from Xenon anaesthesia especially the first 3 hours post extubation are not available. In a study by Coburn et al[27] the post-operative recovery of cognitive function was investigated in elderly patients 65-75 years old without or at a low risk to develop psycho-cognitive dysfunction post-operatively anaesthetized with either Xenon or Desflurane. As a test entity, the test for performance of attention with a subtest of alertness, dividing attention and working memory was used (TAP-test). The tests were performed pre-operatively and 6-12 hours as well as 66-72 hours post-operatively. The tests were performed at the highest difficulty level. At all points in time the result of the TAP-test did not show any statistically significant change between the groups, neither pre-operatively nor post-operatively. Recently, the incidence of psycho-cognitive dysfunction after Xenon or Propofol anaesthesia was also investigated in elderly patients (65-83 years old) by Höcker et al. [28]. In his study the test battery used was based on the neurophysiological tests performed in the "International Studies of Post-Operative Cognitive Dysfunction (POCD) 1 and 2" [29,30]. The tests were realized as baseline 1 day before and subsequently 1 day, 6 days and 30 days after surgery. The incidence of POCD after Xenon or Propofol anaesthesia was only slightly reduced in the Xenon group as a trend at all days post-operatively. The investigation of the incidence of POCD after Xenon or Isoflurane anaesthesia in patients at risk was not the aim of our study. In the present study young and healthy patients were investigated to prove the recovery of memory and attentiveness the first hours following extubation.

No investigations are available as to which one of the following tests are better to cover the early post-operative recovery and which time point is the best to test: The TAP-test, the SST, or f. e. the Mini Mental-test [31]. In the present investigation the SST was used in accordance with Erzigkeit in order to determine the outcome of the variable "cognitive productive efficiency". A more intensive and longer test like the TAP-tests partly shows the extent of mental fatigue over time. In the early post-operative phase mental fatigue after extensive interventions may be due to somatic reasons. With standardization and measuring time to run exercises and registering the number of mistakes by remembering symbols in the authors' opinion the SST is especially appropriate to evaluate the early post-operative course of the cognitive productive efficiency. The individual intelligence quotient of the patients is incorporated in the test system. This is of advantage when compared with the TAP-test. The interpretation of the procedure allows for a statement about the severity of a post-operatively measured disturbance of memory or attentiveness[32,33]. The difference of the blood-gas partition coefficient of the inhalational anaesthetics investigated by Coburn is considerably smaller as compared to the present study. Therefore, the expected difference in the speed of recovery after the end of anaesthesia with either Xenon or Desflurane must be smaller in his study. The first test evaluation post-operatively shows the same result at baseline level and already complete recovery in both groups after 6-12 hours. In the setting of Coburn the time span between extubation and the first measurement of cognitive recovery was too long to expect any difference. In the authors opinion the first testing time was simply too late to discriminate the groups. This fact seems to be more important than the test systems used.

The Aldrete score showed an advantage of Xenon over 45 minutes post extubation. In many cases this time is corresponding to the resting time in the recovery room and may be a further advantage of the Xenon anaesthesia. This was not observed and measured in long and invasive operations like high volume liposuction.

There are some limiting facts of this study. Depending on the kind of surgery and the early post-operative course of treatment the optimal condition that the patient has to have is not defined. It remains unclear if a higher state of attentiveness and better memory of the patients already a few hours post extubation are a real advantage for stationary patients and have any influence on treatment and costs. The resting time in the recovery room, only dependent on the medical condition of the patients was not measured and the effort for care of the staff in the recovery unit and thereafter on the ward was not investigated.

## Conclusion

In summary it has been shown that the wake-up time after anaesthesia with Xenon is faster compared to Isoflurane in N_2_O. Overall, it is concluded that in the referred study the differences between the Xenon and Isoflurane group in the RI, the SST sum score, the sub-scores for memory and attentiveness and the Aldrete score are clinically relevant for the treatment directly after the extubation, the resting time in the recovery room and on the normal ward directly thereafter. A better memory and a higher state of attentiveness may help to facilitate early post-operative care and improve cooperation and compliance of the patients at the day of operation. A relation between the speed of recovery (RI) and the early post-operative cognitive function as the improvement of the SST was shown after Xenon anaesthesia. Further studies will have to elucidate that Xenon anaesthesia combined with other short acting drugs may have an advantage on general outcome post-operatively not only in healthy patients but especially in elderly patients being at risk developing cognitive dysfunction after anaesthesia and surgery.

## Competing interests

The xenon used in the trail was provided by Air Liquide.

## Authors' contributions

RS designed the study, reviewed the literature and drafted the manuscript and the revision of it. JJ and SL performed the anaesthesia, helped to draft the manuscript and took care of the patients. KS performed the SST, UU performed some of the statistical tests and PH helped to draft the reviewed manuscript, controlled the literature, did some statistical tests and was responsible for correspondence and the revision process of the manuscript. All authors read and approved the manuscript.

## Pre-publication history

The pre-publication history for this paper can be accessed here:

http://www.biomedcentral.com/1471-2253/10/5/prepub
